# Safety Assessment of *Antrodia cinnamomea* Cultured in Red Quinoa and Barley: Genotoxicity in Mice and 90-Day Feeding Toxicity in Rats

**DOI:** 10.3390/foods15152723

**Published:** 2026-08-03

**Authors:** Yan-Zhen Dai, Chun-Lin Lee, Jin-Yuarn Lin, Yew-Min Tzeng, Jiunn-Wang Liao

**Affiliations:** 1 Research Center for Animal Medicine, National Chung Hsing University, Taichung 402202, Taiwan; 2 Department of Life Science, National Taitung University, Taitung 950309, Taiwan; 3 Department of Food Science and Biotechnology, National Chung Hsing University, Taichung 402202, Taiwan; 4 Department of Applied Science, National Taitung University, Taitung 950309, Taiwan; 5 Graduate Institute of Veterinary Pathobiology, National Chung Hsing University, Taichung 402202, Taiwan

**Keywords:** *Antrodia cinnamomea* mycelium, genotoxicity, 90-day feeding toxicity

## Abstract

*Antrodia cinnamomea* (AC) is a medicinal mushroom native to Taiwan. This study evaluated the genotoxicity and toxicity of red quinoa and barley-based solid-state fermented AC mycelium (RQBACM). The safety of RQBACM was evaluated via a 90-day repeated-dose oral toxicity study in rats and a battery of genotoxicity assays, including the Ames, CHO-K1 chromosomal aberration, and in vivo micronucleus tests. RQBACM exhibited no mutagenicity in the Ames test, regardless of S9 metabolic activation, nor did it induce chromosomal aberrations in CHO-K1 cells. In mice, no significant changes in reticulocyte or micronucleus frequency were observed at doses up to 5000 mg/kg. In the 90-day study, Sprague–Dawley rats receiving up to 3000 mg/kg/day showed no treatment-related adverse effects, except for a slight reduction in body weight in high-dose males. No significant changes were found in hematology, serum biochemistry, urinalysis, organ weights, or histopathology. The no-observed-adverse-effect level (NOAEL) was determined to be 3000 mg/kg/day, supporting the safety of RQBACM for potential human consumption.

## 1. Introduction

*Antrodia cinnamomea* (AC) belongs to the *Polyporaceae*, *Basidiomycotine* family, and it usually grows on the trunks of *Cinnamomum kanehirai*, a broad-leaved evergreen tree that can be found at altitudes between 450 and 1200 m [1]. The indigenous people of Taiwan use the fruiting bodies of AC to treat abdominal pain and food poisoning and enhance liver function.

Many studies have confirmed that AC mycelium can provide several beneficial effects, including antioxidant, anti-inflammatory, and other biological activities, which can help with various diseases by acting as a liver protectant and producing anti-cancer effects [2,3,4,5]. In particular, AC mycelium in several studies has shown potential for use as an adjuvant therapy for cancer treatment by enhancing radiation and chemotherapy drugs while also reducing the side effects of chemotherapy and the occurrence of resistance [6,7,8,9].

Unfortunately, due to the scarcity of wild AC fruiting bodies, there is a struggle to keep up with market demand. However, with the advancement of biotechnology in recent years, various artificial cultivation techniques of AC have been gradually developed, mainly consisting of four types: submerged fermentation, solid-state culture, cut wood culture, and dish culture [2]. Many studies have confirmed that the different cultivation methods can produce different active components and contents in AC [10,11].

In a recent study comparing different mushroom species cultivated on various cereal substrates, cereal-based solid-state fermentation was shown to significantly enhance mycelial growth, total polyphenol content, and antioxidant activity of the resulting products [12]. Consequently, cereal fermentation has attracted considerable attention as an effective strategy for improving the functional properties of mushroom-derived products. Yang et al. further demonstrated that AC cultivated through cereal-based solid-state fermentation produced higher levels of secondary metabolites, including polyphenols and triterpenoids, than submerged cultivation. These metabolite-rich extracts also exhibited superior antioxidant and anti-tumor activities, highlighting the advantages of solid-state fermentation for enhancing the bioactivity of AC products [13].

Red quinoa (*Chenopodium formosanum* Koidz) is a native cereal of Taiwan that has high nutritional value compared to other grains. It is composed of about 50% starch, 14% dietary fiber, 14% protein, and has a high content of limited essential amino acids, such as lysine [14]. Red quinoa contains a high amount of betanin, which is an indole-containing compound that gives red quinoa its coloration. It not only helps protect the skin by reducing UV damage, but it can also help control body weight [15]. Among cereal substrates, red quinoa has gained increasing interest due to its well-documented nutritional advantages. Previous studies have shown that red quinoa can stimulate the secretion of extracellular enzymes, such as amylase and cellulase, thereby facilitating the release and biosynthesis of bioactive compounds during fermentation [16]. In addition, barley is a rich source of β-glucans and phenolic compounds and has been widely utilized as a functional cereal substrate because of its health-promoting and antioxidant properties [17]. Therefore, the combination of red quinoa and barley may provide a nutritionally favorable matrix for AC growth and metabolite production during solid-state fermentation.

Previous studies have confirmed that the no-observed-adverse effect level (NOAEL) of submerged fermentation and commercial products of AC mycelium in Sprague–Dawley (SD) rats is 3000 mg/kg BW/day and 2800 mg/kg BW/day, respectively [18,19,20]. Although various studies have investigated AC fermentation using cereal substrates, most have focused on single-substrate systems or submerged cultivation. To date, limited information is available regarding the application of a dual-substrate system combining red quinoa and barley for solid-state fermentation of AC, particularly with respect to its safety evaluation. Therefore, the present study is novel in combining these two functional cereal substrates with AC solid-state fermentation and providing a comprehensive toxicological evaluation to support their potential application as functional food ingredients. Accordingly, the present study aimed to evaluate the genotoxic potential and establish the NOAEL of solid-state fermented AC mycelium. To achieve this, a comprehensive toxicological battery was employed, encompassing a bacterial reverse mutation (Ames) test, an in vitro mammalian chromosomal aberration test, and an in vivo mouse peripheral blood micronucleus assay. These findings are intended to furnish critical baseline safety data for human consumption, thereby fostering the commercial and regulatory development of AC.

## 2. Materials and Methods

### 2.1. Test Substance

**Figure 1:**
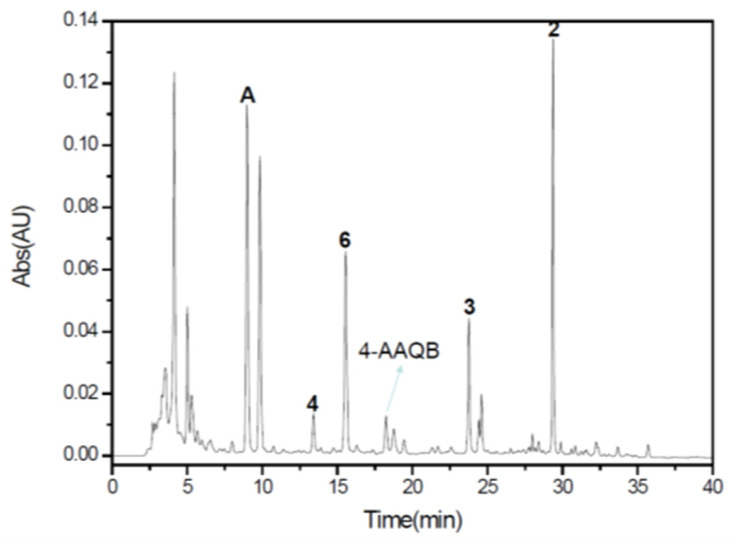
Representative qualitative HPLC chromatogram of RQBACM showing the characteristic triterpenoid constituents. A: 4,7-dimethoxy-5-methyl-1,3-benzodioxole, 2: dehydroeburicoic acid, 3: 15α-acetyl dehydrosulphurenic acid, 4: 3β, 15α-dihydroxylanosta-7,9(11),24-triene-21-oic acid,4-AAQB: 4-acetylantroquinonol B, 6: dehydrosulphurenic acid.

The red quinoa and barley-based solid-state fermented AC mycelium (RQBACM) used in this study was provided by National Taitung University. Antrodia cinnamomea strain NTTU 206, isolated from wild fruiting bodies in Eastern Taiwan, was maintained on potato dextrose agar (PDA) at 30 °C. Seed culture was prepared by transferring agar plugs into potato dextrose broth and incubating at 30 °C with shaking at 150 rpm for 7 days. For solid-state fermentation, sterilized red quinoa and barley substrates were inoculated with the seed culture and incubated at 27 °C under dark conditions with controlled humidity for 90 days. The resulting fermented products were dried and subsequently analyzed for active compounds using HPLC as previously described [21]. To further characterize the chemical profile of RQBACM, HPLC analysis was performed to generate a chromatographic fingerprint of the fermented product. The resulting chromatogram demonstrated a consistent peak pattern, indicating the reproducibility of the fermentation process. This fingerprint serves as a qualitative standard for product characterization and supports the chemical consistency of RQBACM across batches. HPLC analysis was performed using a Hitachi liquid chromatography system (Hitachi, Tokyo, Japan) equipped with an L-7100 pump, autosampler, and L-7400 diode array detector (DAD). Chromatographic separation was achieved on a J’sphere ODS-M80 C18 column (250 × 4.6 mm, 4 μm; YMC, Kyoto, Japan) at room temperature. The mobile phase consisted of acetonitrile (A) and water containing 0.2% acetic acid (B), using a linear gradient from 60:40 to 95:5 (A) over 40 min. The flow rate was maintained at 0.8 mL/min, the injection volume was 5 μL, and detection was performed at 248 nm. UV spectra were simultaneously recorded from 190 to 400 nm for peak characterization [22] (Figure 1). For in vitro assays (Ames test and chromosomal aberration test), the dried fermented RQBACM powder was directly suspended in dimethyl sulfoxide (DMSO) at concentrations of 50 and 200 mg/mL, respectively. The suspensions were thoroughly mixed and centrifuged at 3000 rpm to remove insoluble particulate matter. The resulting supernatant, representing the DMSO-soluble fraction of the crude RQBACM material, was filtered and stored at −20 °C until further use. The ethyl acetate extract was prepared separately for HPLC chemical characterization only and was not used in any biological assays. The RQBACM was suspended in reverse osmosis water for the micronucleus assay and 90-day oral feeding toxicity study. It was freshly prepared before each test.

### 2.2. In Vitro Bacterial Reverse Mutation Assay (Ames Test)

The Ames test was executed in accordance with OECD test guideline No. 471 bacterial reverse mutation test [23], employing five histidine-dependent *Salmonella typhimurium* strains (TA98, TA100, TA102, TA1535, and TA1537). Strains TA98 and TA100 were acquired from the Bioresources Collection and Research Center (BCRC, Hsinchu, Taiwan), whereas TA102, TA1535, and TA1537 were supplied by Discovery Partners International (DPI, San Diego, CA, USA). Following preliminary cytotoxicity assessments to determine appropriate dosing, RQBACM was tested at non-cytotoxic concentrations ranging from 625 to 5000 μg/plate (Table S1).

The assays were performed under both non-activated and metabolically activated conditions. For the latter, a 10% S9 mixture was prepared freshly using an Aroclor 1254-induced rat liver S9 fraction (33.2 mg/mL, Moltox, Boone, NC, USA; Lot# 2901) diluted in a buffer containing 4 μmole nicotinamide adenine dinucleotide phosphate (sodium salt), 5 μmole glucose-6-phosphate (monosodium salt), 8 μmole MgCl_2_, 33 μmole KCl, and 100 μmole sodium phosphate buffer (pH 7.4). For the procedure, 0.1 mL of the bacterial suspension was mixed with 0.2 mL of a 0.5 mM histidine/biotin solution (without S9) or 0.2 mL of the S9 mix (with S9) in top agar, vortexed, and poured onto glucose minimal agar plates. The plates were allowed to harden, inverted, and incubated at 37 °C for 48 h.

Negative (vehicle) controls and specific positive control mutagens were included in each assay. In the absence of S9, positive controls comprised 2.5 μg/plate 4-nitroquinoline-N-oxide (4-NQO) for TA98, 5 μg/plate sodium azide for TA100 and TA1535, 0.5 μg/plate mitomycin C for TA102, and 50 μg/plate 9-aminoacridine (9-AA) for TA1537. In the presence of S9, 2-aminoanthracene (2-AA) at 5 μg/plate was applied to all tester strains. Each concentration in the Ames test was tested using triplicate plates, and the results are expressed as mean ± standard deviation (SD). A positive mutagenic result was defined as a clear dose-dependent, minimum twofold increase in the mean number of revertant colonies compared to the negative control group.

### 2.3. In Vitro Chromosome Aberration Assay

The potential of RQBACM to trigger structural and numerical chromosome damage was investigated via an in vitro chromosomal aberration assay utilizing Chinese hamster ovary K1 (CHO-K1) cells. The methodology strictly adhered to the regulatory guidelines for the safety assessment of health foods. Sourced from the BCRC (Hsinchu, Taiwan), the cells were propagated in Ham’s F-12 medium (Gibco, Grand Island, NY, USA) containing 10% fetal bovine serum (FBS) under standard atmospheric conditions (37 °C and 5% CO_2_). A cell viability test was conducted to determine the highest concentration that caused less than 70% cytotoxicity (Figure S1). Accordingly, a final concentration of 2000 μg/mL RQBACM was introduced into the culture medium for a 24 h exposure. For the non-activated test system, mitomycin C (2.5 μg/mL) was employed as the reference mutagen. Conversely, cyclophosphamide (25 μg/mL) was applied as the positive control for the metabolically activated conditions supplemented with the S9 fraction. Following a 12 h attachment period after seeding CHO-K1 cells (2 × 10^5^ cells/mL) in T25 flasks, the culture supernatant was exchanged for fresh medium supplemented with 1% sample without S9 metabolic activation. The cultures were then maintained under these treatment conditions for 24 h. For S9 fraction incubation, after co-incubation with 1% sample and S9 mixture for 3 h, the medium was refreshed and incubated for 24 h. Colcemid (10 μg/mL) was added 6 h before harvesting. The CHO-K1 cells were harvested and treated with 0.6% KCl to swell the cells. They were then fixed with methanol–acetate (3:1), dropped on a glass slide and stained with Diff-Quik. Chromosomal damage was quantified by evaluating at least 300 well-spread metaphases for each treatment level. The overall aberration rate (%) was determined according to the following formula:

$$Aberration\;rate\;(\%)=\frac{Number\;of\;cells\;with\;aberrant\;chromosomes}{Total\;number\;of\;examined\;cells}\times 100$$

Criteria for a positive mutagenic response required a distinct, minimum twofold elevation in chromosomal aberration frequency compared with the vehicle control baseline.

### 2.4. In Vivo Peripheral Blood Micronucleus Assay in Mice

For the in vivo micronucleus assay, 5-week-old male ICR mice were obtained from BioLASCO Taiwan Co., Ltd. (Yilan, Taiwan) and acclimated for 5 days. Animals were housed five per cage under controlled conditions (21 ± 2 °C, 50–70% humidity, and a 12 h light/dark cycle) with free access to food (LabDiet 5001; Purina Mills LLC, St. Louis, MO, USA) and water. The study protocol was officially authorized by the IACUC of National Chung Hsing University (No. 107-132, approval date: 5 December 2018). Following the official health food safety assessment guidelines, 25 mice were randomly allocated into five groups (*n* = 5 per group) based on initial body weights. The negative and positive controls received an oral gavage of water (20 mL/kg) and an intraperitoneal injection of cyclophosphamide (60 mg/kg), respectively. The three treatment groups were orally administered RQBACM at doses of 1000, 3000, or 5000 mg/kg BW at a volume of 20 mL/kg. Animals were monitored daily for morbidity and mortality, with moribund mice scheduled for immediate euthanasia.

Under 2% isoflurane anesthesia (Halocarbon Laboratories, Peachtree Corners, GA, USA), 100 μL of peripheral blood was sampled at 48 and 72 h post-dosing. Blood smears were prepared and stained with 0.1% acridine orange, followed by evaluation under a fluorescence microscope (BX50; Olympus, Münster, Germany), where RETs and MN appeared orange and yellow-green, respectively. Genotoxicity was quantified by scoring 1000 RETs per animal to determine the frequency of MN-RETs. Bone marrow toxicity was concurrently indexed by checking the RET/NCE ratio against 1000 counted NCEs. Ultimately, a positive mutagenic outcome was defined as a statistically significant, dose-dependent surge in the MN-RET frequency compared with the vehicle control baseline.

### 2.5. In Vivo 90-Day Oral Feeding Toxicity Study in Rats

Following OECD test guideline 408 repeated dose 90-day oral toxicity study in rodents [24], a 90-day subchronic oral toxicity evaluation was conducted using 5-week-old Sprague Dawley rats (BioLASCO, Yilan, Taiwan). Under IACUC protocol 107-070 (approval date: 28 June 2018), 80 rats (10 per sex/group) were randomly allocated to receive either RO water (control) or RQBACM at daily oral gavage doses of 1000, 2000 or 3000 mg/kg. Throughout the study, clinical symptoms, mortality, body weight, and food intake were systematically monitored. On day 91, fasted rats were anesthetized with 2% isoflurane to facilitate blood collection from the abdominal aorta. Standard hematology, serum chemistry, and urinalysis were performed using Sysmex XE2100 (Sysmex, New Taipei, Taiwan), ADVIA 1800 (Siemens Healthineers, Erlangen, Germany), and Clinitek 100 systems (Siemens Healthineers, Erlangen, Germany), respectively.

A comprehensive necropsy was performed on all subjects, where organs were excised, weighed, and fixed in 10% buffered formalin. Histopathological examination was conducted on a full tissue battery (including respiratory, digestive, endocrine, and reproductive systems) from the control and 3000 mg/kg groups. The severity of identified lesions was scored on a 5-point scale (1: minimal, <10%; 2: slight, 11–25%; 3: moderate, 26–50%; 4: moderate/severe, 51–75%; 5: severe, 76–100%) as per Shackelford et al.’s [25] methodology.

### 2.6. Statistical Analysis

Statistical analysis was performed using SPSS software (Version 31.0; IBM Corp., Armonk, NY, USA). All continuous data were expressed as mean ± SD. One-way analysis of variance (ANOVA) was used to assess differences among groups, followed by Dunnett’s post hoc test for multiple comparisons between each treatment group and the control group. In addition, the Jonckheere–Terpstra trend test was applied to evaluate dose-dependent trends across ordered treatment groups. For incidence or categorical data, Fisher’s exact test was used. A *p*-value of less than 0.05 was considered statistically significant.

## 3. Results

### 3.1. Ames Test

The results of bacterial toxicity tests conducted before the Ames test showed that the RQBACM was not toxic to *Salmonella* strains TA98, TA100, or TA1535 at 5000 μg/plate, but was toxic to TA102 and TA1537 at 1250 μg/plate (Table S1). In the Ames test, the mean background revertant colony counts for the five tester strains in the negative control groups fell within historical control ranges, both in the presence and absence of S9 metabolic activation. The positive controls for the 5 test strains induced more than a two-fold increase, ranging from 2- to 96-fold, and were significantly increased when compared to the negative control group. Across all evaluated concentrations of RQBACM, revertant colony counts demonstrated no significant increase (*p* > 0.05) under both metabolically active and inactive conditions (Table 1). The Ames test profiles thus demonstrate that RQBACM is devoid of mutagenic potential toward the tested *Salmonella* strains.

### 3.2. Chromosome Aberration Assay

The IC_70_ of RQBACM was identified as 2000 μg/mL following a 24 h exposure period with CHO-K1 cells (Figure S1). This concentration was subsequently utilized as the maximum dosage for the chromosomal aberration assay. The results of the chromosomal aberration assay are shown in Table 2. The historical control range for spontaneous chromosomal aberrations in CHO-K1 cells in our laboratory is 2.7–5.7% (Table S2). The observed values in the present study (1.7–4.0%) are generally consistent with this range, with minor variation considered within normal biological and experimental variability. In the positive control groups, mitomycin C and cyclophosphamide significantly increased the percentage of chromosomal aberrations (*p* < 0.05) for coincubation with or without S9 fraction when compared to the negative control group. RQBACM at all evaluated concentrations (500, 1000, and 2000 μg/mL) exhibited no statistically significant increase (*p* > 0.05) in chromosomal aberration frequency, both in the presence and absence of S9 metabolic activation (Table 2). No effects on chromosome aberration were observed in CHO-K1 cells after RQBACM incubation with or without S9 metabolic activation.

### 3.3. In Vivo Micronucleus Assay in Mice

No mortality or treatment-related clinical abnormalities were recorded, and body weight patterns remained unaffected during the study (Table S3). The comprehensive micronucleus profiles evaluated at 48 and 72 h post-dosing are presented in Table 3. As anticipated, the positive control group demonstrated a significant reduction in the RET proportion (*p* < 0.05) alongside a concurrent surge in the MN frequency (*p* < 0.05) compared with the vehicle control at both sampling intervals (Table 3). These outcomes demonstrate that RQBACM is devoid of bone marrow toxicity and genotoxic potential, as evidenced by the lack of erythropoiesis inhibition or micronuclei induction.

### 3.4. The 90-Day Oral Toxicity Study in Rats

#### 3.4.1. Survival, Clinical Observation, Body Weight and Feed Consumption

**Figure 2:**
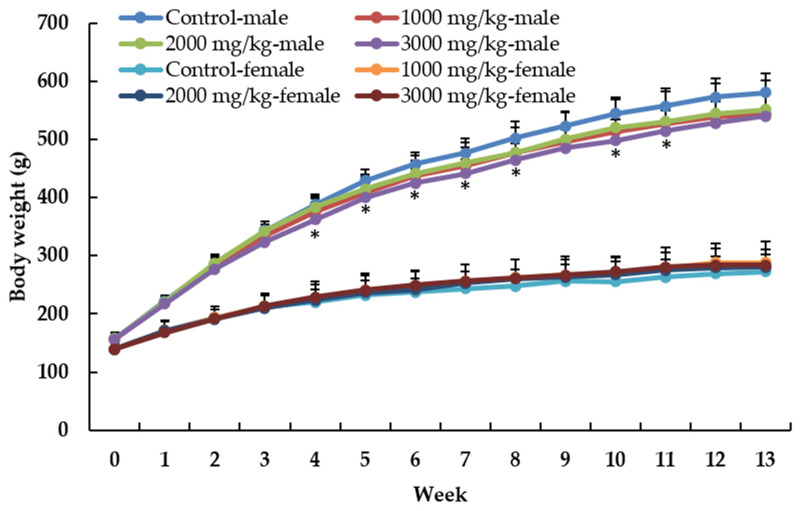
Body weight change in rats administered RQBACM in the 90-day feeding toxicity study. * Significant difference between the control and male high-dose group at *p* < 0.05.

**Figure 3:**
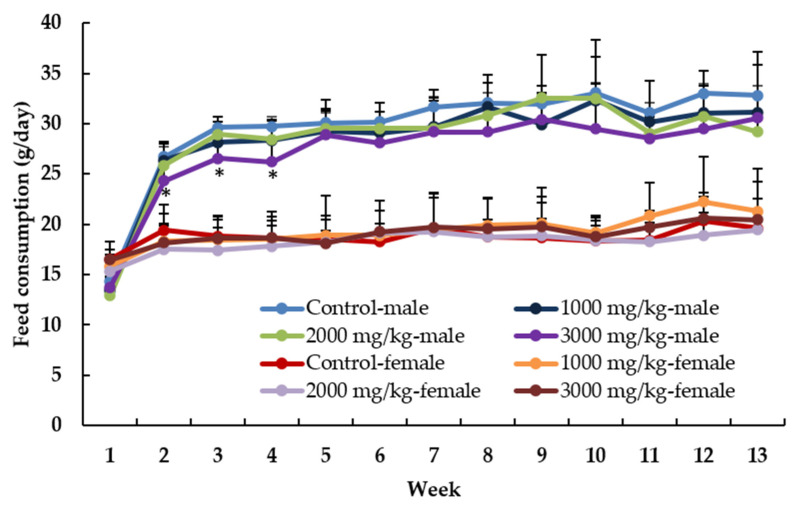
Feed consumption of rats administered RQBACM in the 90-day feeding toxicity study. * Significant difference between the control and high-dose groups of male rats at *p* < 0.05.

No observable toxic signs or clinical abnormalities occurred in the rats across all RQBACM dosage groups over the course of the study. The death of a single male rat in the middle group was attributed to gavage error. Starting from week 4 to week 8 and week 10 to week 11, male rats in the high-dose cohort exhibited a statistically significant reduction in body weight gain relative to the control group (*p* < 0.05, Figure 2). This suppressed weight accumulation closely paralleled a prominent decline in weekly food intake; specifically, feed consumption in high-dose males was significantly lower than that of controls during weeks 2–4 (*p* < 0.05, Figure 3). There was no significant difference observed in terms of feed efficiency of male or female rats in the treated groups (Figure S2).

#### 3.4.2. Hematology

Hematological profiling revealed that white blood cell counts were entirely unaffected by treatment in both male and female rats. A statistically significant decrease in fibrinogen levels was observed in low-dose females (*p* < 0.05, Table 4). Because this significant difference occurred sporadically and lacked dose-dependency, this was interpreted as spontaneous biological variations rather than test-article-induced adverse effects.

#### 3.4.3. Serum Chemistry

As summarized in Table 5, repeated-dose exposure to the high dose of RQBACM induced several isolated alterations in serum biochemistry. High-dose males exhibited significantly increased levels of sodium (*p* < 0.05) compared with the vehicle controls. In contrast, statistically significant increases in uric acid and potassium levels were observed in high-dose females compared with the control group (*p* < 0.05). Because this statistical difference was devoid of any physiological anomalies and fell entirely within normal physiological baselines (male sodium: 121.9–162.6 mEq/dL; female potassium: 2.2–5.4 mEq/dL), they were classified as spontaneous biological variations rather than treatment-related adverse effects.

#### 3.4.4. Urinalysis

Comprehensive urinalysis conducted on Day 0 and Day 90 revealed no treatment-related toxicological abnormalities in either sex across all RQBACM groups compared to controls. Evaluated parameters encompassed physical and chemical properties (volume, color, specific gravity, pH, protein, glucose, bilirubin, ketones, and nitrite) as well as microscopic sediment examinations (erythrocytes, leukocytes, urinary casts, crystals, and parasites), all of which remained within normal physiological ranges.

#### 3.4.5. Necropsy, Organ Weight and Histopathology

Terminal pathological assessments, comprising macroscopic and microscopic evaluations, were executed following the scheduled sacrifice of all animals. Gross pathological scoring detected localized abnormalities in a minority of subjects: mid-dose males displayed bilateral testicular atrophy (incidence: 1/9) and multifocal lung erythema (incidence: 1/9), while a single high-dose female presented with left kidney enlargement (incidence: 1/10) (Table S4). The test article did not significantly affect the absolute weights of major organs (Table 6). Although a statistically significant increase in relative liver weight was observed in high-dose males, a dose-dependent trend was also noted (*p* < 0.05, Table 7). However, no corresponding changes were observed in serum liver function parameters or liver histopathological findings. Considering that the change in relative liver weight may have been influenced by differences in terminal body weights among groups, this finding was not considered to represent a treatment-related toxic effect of RQBACM. Microscopic examination conclusively established that none of these gross lesions or organ weight variations were test-article-induced. In some rats, focal infiltration of mononuclear inflammatory cells was observed in the Harderian gland, heart, and prostate. In the kidney, lesions including focal tubular cystic dilatation, mononuclear inflammatory cell infiltration, tubular regeneration, and pyelonephritis were noted. In the pancreas, focal ductal atrophy, fibrosis and pigment deposition in the islets of Langerhans and mononuclear inflammatory cell infiltration were observed. One male rat in the mid-dose group exhibited pulmonary lesions due to accidental intratracheal administration during gavage, resulting in multifocal hemorrhage with histopathological findings consistent with foreign-body obstruction containing test substance. Another male rat in the mid-dose group showed bilateral cryptorchidism, with histopathology revealing diffuse seminiferous tubule atrophy, a commonly observed congenital lesion in rats. A pulmonary red spot was observed in one male rat in the mid-dose group, which showed no corresponding histopathological abnormalities. In one female rat in the high-dose group, enlargement of the left kidney was observed, with histopathological findings of diffuse pyelonephritis, urinary bladder mononuclear inflammatory cell infiltration, and epithelial hyperplasia. These lesions showed no dose-dependent relationship in either incidence or severity when compared with the control and high-dose groups and were therefore considered nonspecific and unrelated to treatment. Overall, histopathological examination demonstrated that oral administration of RQBACM at the highest dose (3000 mg/kg body weight) for 90 consecutive days did not induce treatment-related toxicologically significant pathological changes in major organs of male or female rats (Table S5).

## 4. Discussion

Mutations that occur in oncogenic or tumor suppressor genes in somatic cells, such as point mutations, can cause tumors in humans or experimental animals, and the Ames test is often used to detect whether test articles can cause genetic mutations. In the previous study, the results of solid-state cultured AC mycelium in the dose-range determination test conducted before the Ames test showed no bacterial toxicity to *Salmonella typhimurium* strains at 5000 μg/plate. However, RQBACM significantly inhibited bacterial growth at 1250 μg/plate. Although RQBACM showed inhibitory effects against *Salmonella* toxicity assay, it is not sufficient to directly conclude that it possesses stronger antibacterial activity than submerged–fermented AC mycelium. This observation may be related to differences in fermentation systems, as solid-state fermentation has been reported to enhance the production of bioactive secondary metabolites and antimicrobial activities compared to submerged fermentation [26]. In addition, the red quinoa substrate may contribute additional functional compounds that could influence the observed effects [27]. The Ames test utilizing *Salmonella typhimurium* strains demonstrated that solid-state cultured AC mycelium possessed no mutagenic activity, either in the presence or absence of S9 metabolic activation. Crucially, the validity and sensitivity of the test system were confirmed by the concurrent positive controls, which consistently elicited a greater than twofold increase in the number of revertant colonies under both metabolic conditions. The results of the Ames test did not indicate any genotoxicity, which is consistent with previous studies [19,28].

Several studies have confirmed that aberrations in chromosome structure and number are associated with many diseases, including congenital diseases and cancers [29]. The in vitro mammalian cell chromosome aberration test is used to identify whether test substances cause mutations in chromosomal structure and to screen for possible mutations and substances that are carcinogenic to humans. The in vitro mammalian chromosomal aberration test in the present study demonstrated that the test substance possessed no clastogenic activity, showing no significant increase in the frequency of structural chromosomal aberrations either in the presence or absence of S9 metabolic activation. In contrast, the concurrent positive controls elicited the expected significant increases in chromosome abnormalities, confirming the reliability of the assay. These findings align with prior documented literature [19,28].

To assess in vivo cytogenetic damage in mammals, the micronucleus test monitors the induction of small cellular structures that harbor lagging chromosomal fragments or intact chromosomes excluded from the main nucleus during defective cell division. This damage may be induced by the test chemical, and it effects the chromosomes or the mitotic apparatus of erythroblasts. In the micronucleus test, no clinical symptoms or significant weight changes were observed, and the numbers of RETs and Mn-RETs (‰) were consistent with the results in previous studies [19,28]. This indicated that RQBACM would not cause erythrocyte chromosome mutations or damage during mitosis. Although having five animals per group means that the minimum requirement of OECD Guideline 474 is met for the in vivo micronucleus test, a larger group size is generally recommended to improve statistical power. Therefore, the relatively small number of animals used in this study may be considered a limitation.

To explore the adverse effects of the long-term consumption of RQBACM, the 90-day feeding oral toxicity test in SD rats was conducted according to OECD guideline 408. The results of this experiment showed that the body weights of male rats in the high-dose groups were significantly decreased compared with those of the control group during Weeks 4–8 and Weeks 10–11. However, the body weights of the high-dose group of male rats increased steadily during the experiment. In a similar case, lower weights were also observed in the high-dose male rats and all treatment groups in female rats when compared to the control group [18]. Furthermore, in a teratogenicity study in 2014, oral administration of 1500 to 3500 mg/kg BW AC mycelium caused the feed consumption of pregnant female rats to decrease by 7.2 to 12.6% when compared with the control group. One possible reason is that when the rats were given a large amount of AC mycelium, it decreased the feed intake [30]. In a 28-day feeding toxicity study, it was found that the body weights of the high-dose male rats were lower than the control group, probably due to improper tube feeding of the animals, which reduced feed intake [5]. The reason that the feed intake of male rats in the high-dose group in this experiment was lower than that of the control group may be that the large amount of AC mycelium caused a higher intake of dietary fiber. The literature indicates that without restricting caloric intake, increasing dietary fiber intake can help with weight loss, especially for obese individuals in which the effect of weight loss is more significant [31]. In the present experiment, AC mycelium was fermented with red quinoa and barley as a solid substrate. It has been suggested that this higher proportion of fiber, which increases satiety after administration of RQBACM, reduces feed intake and affects weight change. In addition, the literature has pointed out that red quinoa can inhibit fat accumulation and adipogenesis in 3T3-L1 mouse preadipocytes [32]. The active ingredients of red quinoa that can reduce fat as stated in the literature need to be explored. Furthermore, AC mycelium contains amino acids, which may have a bitter taste and may reduce appetite in rats [33]. Although transient reductions in body weight were observed in high-dose male rats, the magnitude of the decrease remained below 10% throughout the study and was not accompanied by reduced feed efficiency or treatment-related clinical, biochemical, or histopathological changes. Therefore, these findings were considered to be of limited toxicological significance and were not regarded as adverse effects. However, a limitation of the present study is that certain endpoints introduced in the 2018 revision of OECD Test Guideline 408, including functional observational battery assessments, ophthalmological examinations, and thyroid hormone measurements, were not included because the study protocol had been finalized before implementation of the revised guideline.

## 5. Conclusions

In conclusion, RQBACM showed no genotoxic potential and no treatment-related adverse effects in a 90-day subchronic oral toxicity study. Consequently, the no-observed-adverse-effect level (NOAEL) for RQBACM in both male and female rats was firmly established at 3000 mg/kg BW/day under the experimental conditions evaluated.

## Figures and Tables

**Table 1:** Results of RQBACM in *Salmonella typhimurium* TA98, TA100, TA1535, TA 102, and TA1537 mutagenicity test.

Group	Number of Revertant (Colony/Plate)				
	TA98	TA100	TA1535	TA102	TA1537
Without S9 metabolic activation					
Negative ^1^	40.7 ± 2.9 ^4^	167.0 ± 8.6	12.7 ± 3.1	269.7 ± 7.6	11.3 ± 3.1
Positive ^2^	271.0 ± 43.5	2630.0 ± 461.9	1220.0 ± 115.2	1293.3 ± 47.8	581.7 ± 13.3
RQBACM ^3^ (μg/plate)					
39.1	-	-	-	263.0 ± 3.7	9.7 ± 0.5
78.1	-	-	-	263.3 ± 7.7	9.0 ± 5.0
156.3	-	-	-	258.3 ± 5.3	9.3 ± 2.1
312.5	46.0 ± 1.6	169.0 ± 2.9	10.7 ± 3.3	261.7 ± 4.2	6.7 ± 0.9
625	43.7 ± 3.1	175.0 ± 8.6	13.7 ± 2.1	272.3 ± 2.5	9.3 ± 1.2
1250	45.3 ± 1.7	176.7 ± 7.1	14.7 ± 2.5	-	-
2500	42.7 ± 0.5	180.3 ± 5.4	14.3 ± 4.0	-	-
5000	35.7 ± 8.2	172.0 ± 0.8	16.0 ± 0.8	-	-
With S9 metabolic activation					
Negative	41.3 ± 3.3	186.0 ± 7.9	15.7 ± 1.7	390.3 ± 8.2	8.0 ± 0.8
Positive	2140.3 ± 735.5	560.3 ± 50.1	292.7 ± 33.4	1043.3 ± 49.2	158.7 ± 6.1
RQBACM (μg/plate)					
39.1	-	-	-	385.7 ± 5.4	9.7 ± 1.7
78.1	-	-	-	377.7 ± 9.3	7.7 ± 0.5
156.3	-	-	-	392.7 ± 3.9	13.0 ± 3.6
312.5	35.7 ± 4.2	186.7 ± 9.5	11.7 ± 2.4	385.3 ± 5.2	10.0 ± 2.2
625	41.3 ± 6.2	193.3 ± 6.6	12.3 ± 5.2	389.0 ± 6.2	9.3 ± 0.5
1250	37.7 ± 1.7	184.3 ± 7.1	16.3 ± 2.9	-	-
2500	39.7 ± 5.4	178.3 ± 4.1	17.3 ± 3.1	-	-
5000	37.7 ± 3.3	174.0 ± 6.7	16.0 ± 3.7	-	-

^1^ Negative control: DMSO. ^2^ Positive controls (in the absence of S9 metabolic activation): 4-nitroquinoline-N-oxide (2.5 μg/plate) for TA98, sodium azide (5 μg/plate) for TA100 and TA1535, mitomycin C (0.5 μg/plate) for TA102, and 9-aminoacridine (50 μg/plate) for TA1537. Positive control (in the presence of S9 metabolic activation): 2-aminoanthracene (5 μg/plate) for all *Salmonella typhimurium* tester strains. ^3^ RQBACM: red quinoa and barley-based solid-state fermented AC mycelium ^4^ Data are expressed as mean ± SD (*n* = 3).

**Table 2:** Frequency of chromosomal aberrations in CHO-K1 cells treated with RQBACM.

Group	Total Aberrations	Frequency of Chromosomal Aberration (%) ^1^
Without S9 metabolic activation		
Negative control	12/300	4.0 ± 1.7 ^2^
Mitomycin C (2.5 μg/mL)	89/300	29.7 ± 3.5 *
RQBACM (μg/mL)		
500	9/300	3.0 ± 2.6
1000	19/300	6.3 ± 0.6
2000	15/300	5.0 ± 1.0
With S9 metabolic activation		
Negative control	5/300	1.7 ± 2.1
Cyclophosphamide (25 μg/mL)	68/300	22.7 ± 6.0 *
RQBACM (μg/mL)		
500	10/300	3.3 ± 1.5
1000	10/300	3.3 ± 4.2
2000	12/300	4.0 ± 3.5

RQBACM: red quinoa and barley-based solid-state fermented AC mycelium. ^1^ Following a three-step Diff-Quik staining, 100 metaphases per plate (300 per dose) were microscopically analyzed. Results represent the total aberrations per treatment. ^2^ Cells with structural chromosomal damage were counted to calculate the percentage of aberrant cells: Aberration rate (%) = (number of cells with aberrant chromosomes/total number of examined cells) × 100. Data are presented as mean ± SD (*n* = 3). * denotes a significant difference relative to the negative control group (*p* < 0.05).

**Table 3:** Effects of RQBACM oral administration on micronucleus induction in peripheral blood reticulocytes of male mice.

Group/	Dose (mg/kg)	RETs/RBCs (‰)	Mn-RETs/RETs (‰)
Intervals			
Male			
48 h			
NC ^2^	0	36.0 ± 1.8 ^1^	3.2 ± 1.3
PC ^3^	60	10.4 ± 0.8 *	22.8 ± 1.5 *
RQBACM	1000	33.2 ± 2.5	1.8 ± 1.2
	3000	32.8 ± 1.2	2.6 ± 1.5
	5000	35.4 ± 3.9	4.2 ± 1.2
72 h			
NC	0	43.0 ± 3.0	1.8 ± 0.7
PC	60	13.4 ± 1.6 *	16.0 ± 4.3 *
RQBACM	1000	39.2 ± 6.4	1.8 ± 1.2
	3000	40.8 ± 3.7	1.8 ± 0.7
	5000	41.2 ± 4.9	2.2 ± 0.4

^1^ Values represent the mean ± SD (*n* = 5). ^2^ Negative control: distilled water (20 mL/kg BW); ^3^ positive control: cyclophosphamide (60 mg/kg BW, i.p.). * Significantly different from the concurrent negative control at *p* < 0.05. NC, negative control; RETs, reticulocytes; RQBACM: red quinoa and barley-based solid-state fermented AC mycelium; RBCs, erythrocytes; MN-RETs, micronucleated reticulocytes.

**Table 4:** Effects of 90-day repeated-dose oral toxicity study of RQBACM on hematological profiles in rats.

Parameter	Units	Male				Female			
			RQBACM (mg/kg)				RQBACM (mg/kg)		
		Control	1000	2000 ^2^	3000	Control	1000	2000	3000
RBC ^3^	10^6^/μL	9.0 ± 0.3 ^1^	9.0 ± 0.5	9.0 ± 0.3	9.0 ± 0.4	8.4 ± 0.4	8.4 ± 0.3	8.5 ± 0.5	8.4 ± 0.6
HGB	g/dL	16.2 ± 0.4	16.2 ± 0.7	15.8 ± 0.6	16.1 ± 0.5	15.6 ± 0.7	15.5 ± 0.4	15.9 ± 1.1	15.7 ± 0.7
HCT	%	49.8 ± 1.5	49.9 ± 2.4	49.2 ± 1.8	50.1 ± 1.3	47.6 ± 1.9	47.1 ± 1.2	48.3 ± 3.9	48.4 ± 2.5
MCV	fL	55.1 ± 1.4	55.4 ± 1.4	54.7 ± 1.5	55.9 ± 1.9	56.6 ± 1.4	55.9 ± 1.0	56.7 ± 1.5	57.5 ± 1.7
MCH	pg	17.9 ± 0.5	18.0 ± 0.6	17.6 ± 0.5	18.0 ± 0.6	18.6 ± 0.5	18.4 ± 0.4	18.6 ± 0.4	18.6 ± 0.8
MCHC	g/dL	32.5 ± 0.4	32.4 ± 0.4	32.2 ± 0.8	32.2 ± 0.2	32.8 ± 0.5	32.9 ± 0.5	32.9 ± 0.5	32.4 ± 0.4
PLT	10^3^/μL	1044.7 ± 372.2	1186.0 ± 123.7	1098.1 ± 234.7	1224.1 ± 197.9	982.5 ± 272.2	877.8 ± 325.1	938.8 ± 316.2	905.0 ± 261.8
WBC	10^3^/μL	7.8 ± 2.1	7.8 ± 3.3	6.7 ± 2.5	7.5 ± 1.7	4.9 ± 2.2	5.0 ± 1.8	4.3 ± 1.9	5.1 ± 2.2
LYMPH	%	79.4 ± 7.0	83.7 ± 4.2	78.4 ± 7.8	82.8 ± 6.2	81.8 ± 6.8	81.9 ± 6.2	78.7 ± 7.6	77.9 ± 11.4
NEUT	%	17.2 ± 5.5	13.4 ± 3.8	18.1 ± 7.3	14.3 ± 6.3	14.9 ± 6.7	14.8 ± 5.9	17.2 ± 6.6	17.9 ± 10.3
MONO	%	1.5 ± 2.1	0.9 ± 0.2	0.9 ± 0.4	1.4 ± 2.1	1.0 ± 0.3	0.9 ± 0.2	1.1 ± 0.3	1.1 ± 0.6
EOSIN	%	1.8 ± 1.2	1.9 ± 1.1	2.5 ± 2.9	1.4 ± 0.6	2.2 ± 1.1	2.4 ± 1.1	2.9 ± 2.2	3.1 ± 2.7
BASO	%	0.1 ± 0.1	0.1 ± 0.1	0.1 ± 0.1	0.0 ± 0.1	0.1 ± 0.1	0.0 ± 0.1	0.0 ± 0.1	0.1 ± 0.1
PT	sec	11.2 ± 0.8	10.5 ± 0.4	11.6 ± 1.2	11.2 ± 0.8	9.7 ± 0.7	9.9 ± 0.6	9.9 ± 0.3	9.8 ± 0.4
APTT	sec	36.9 ± 4.5	34.0 ± 11.1	34.9 ± 5.3	30.7 ± 5.3	27.1 ± 4.3	28.1 ± 5.5	28.5 ± 5.0	25.5 ± 2.8
FBG	mg/dL	177.1 ± 19.1	192.5 ± 18.5	179.4 ± 16.3	187.7 ± 8.8	139.7 ± 23.6	120.5 ± 10.8 *	140.7 ± 12.5	137.9 ± 19.5

RQBACM: red quinoa and barley-based solid-state fermented AC mycelium ^1^ Data are presented as the mean ± SD (*n* = 10). ^2^ Data are presented as the mean ± SD (*p* = 9). ^3^ Abbreviations: RBC, red blood cell; HGB, hemoglobin; HCT, hematocrit; MCV, mean corpuscular volume; MCH, mean corpuscular hemoglobin; MCHC, mean corpuscular hemoglobin concentration; PLT, platelet; WBC, white blood cell; LYMPH, lymphocyte; NEUT, neutrophil; MONO, monocyte; EOSIN, eosinophil; BASO, basophil. Coagulation: PT, prothrombin time; APTT, activated partial thromboplastin time; FBG, fibrinogen. * Statistically significant difference relative to the control group (*p* < 0.05).

**Table 5:** Serum biochemical parameters of rats in the 90-day repeated-dose oral toxicity study of RQBACM.

Parameter	Units	Male				Female			
			RQBACM (mg/kg)				RQBACM (mg/kg)		
		Control	1000	2000 ^2^	3000	Control	1000	2000	3000
AST ^3^	U/L	72.7 ± 12.1 ^1^	69.7 ± 7.5	77.7 ± 11.0	69.0 ± 9.0	72.5 ± 6.2	83.0 ± 20.2	80.2 ± 25.7	72.9 ± 12.8
ALT	U/L	27.6 ± 3.0	25.9 ± 3.4	26.1 ± 5.0	25.9 ± 4.0	25.4 ± 5.0	35.8 ± 15.1	29.4 ± 13.5	27.7 ± 5.9
ALP	U/L	127.5 ± 156.9	78.2 ± 13.7	81.1 ± 25.3	73.7 ± 10.1	43.3 ± 8.1	45.7 ± 11.0	50.0 ± 14.9 ^2^	50.0 ± 16.4
BUN	mg/dL	16.9 ± 4.8	17.0 ± 1.7	17.3 ± 2.6	15.9 ± 0.9	17.7 ± 3.7	16.0 ± 3.5	17.3 ± 3.1	18.1 ± 4.5
CK	U/L	140.2 ± 57.2	134.7 ± 41.9	161.9 ± 70.1	135.9 ± 41.3	107.0 ± 45.1	105.2 ± 65.8	132.1 ± 101.0 ^2^	85.3 ± 16.0
Creatinine	mg/dL	0.4 ± 0.3	0.3 ± 0.0	0.3 ± 0.1	0.3 ± 0.0	0.3 ± 0.1	0.3 ± 0.0	0.3 ± 0.1	0.3 ± 0.1
Glucose	mg/dL	206.2 ± 20.9	200.9 ± 45.6	212.2 ± 35.5	211.0 ± 42.9	150.8 ± 24.4	143.1 ± 25.2	154.0 ± 25.4 ^2^	135.9 ± 18.4
Cholesterol	mg/dL	45.3 ± 16.0	53.4 ± 9.7	49.1 ± 8.2	55.6 ± 13.5	57.7 ± 7.2	53.9 ± 11.8	63.1 ± 14.2	59.8 ± 9.8
GGT	U/L	<1	<1	<1	<1	<1	<1	<1	<1
LDH	U/L	206.6 ± 150.3	221.1 ± 134.4	248.9 ± 153.4	220.8 ± 107.2	158.6 ± 90.1	169.8 ± 123.9	193.6 ± 188.0	96.2 ± 33.8
TB	mg/dL	<0.1	<0.1	<0.1	<0.1	<0.1	<0.1	<0.1	<0.1
UA	mg/dL	1.4 ± 0.3	1.7 ± 1.1	1.4 ± 0.4	1.8 ± 0.6	1.3 ± 0.4	1.3 ± 0.3	1.2 ± 0.2	1.1 ± 0.2 *
Amylase	U/L	1597.4 ± 123.6	1647.0 ± 230.8	1675.7 ± 206.1	1579.5 ± 176.2	1194.7 ± 158.6	1147.8 ± 335.2	1159.2 ± 167.5	1088.3 ± 240.7
Albumin	g/dL	3.6 ± 0.3	3.7 ± 0.2	3.7 ± 0.2	3.7 ± 0.2	4.0 ± 0.3	4.1 ± 0.4	4.0 ± 0.3	4.1 ± 0.2
HDL-C	mg/dL	9.9 ± 3.3	11.3 ± 2.7	11.1 ± 1.4	11.0 ± 2.4	18.7 ± 2.5	17.6 ± 3.9	20.3 ± 4.5	20.1 ± 3.3
TP	g/dL	5.4 ± 0.3	5.7 ± 0.3	5.6 ± 0.4	5.8 ± 0.4	5.8 ± 0.4	5.9 ± 0.5	5.9 ± 0.3	5.9 ± 0.2
TG	mg/dL	43.4 ± 10.2	45.6 ± 16.5	42.8 ± 13.7	39.5 ± 9.1	37.2 ± 10.8	31.5 ± 6.3	33.7 ± 5.7	28.2 ± 5.5
Ca^2+^	mg/dL	9.3 ± 0.6	9.7 ± 0.4	9.5 ± 0.5	9.8 ± 0.5	9.6 ± 0.3	9.5 ± 0.2	9.6 ± 0.4	9.5 ± 0.3
Cl^−^	mEq/dL	104.6 ± 2.9	104.2 ± 1.7	104.1 ± 2.7	104.5 ± 2.1	106.5 ± 1.2	106.4 ± 1.8	107.0 ± 2.3	106.6 ± 1.4
K^+^	mEq/dL	5.8 ± 1.1	5.7 ± 0.9	5.3 ± 0.7	5.2 ± 0.7	5.1 ± 0.4	5.0 ± 0.4	4.9 ± 0.5	4.5 ± 0.4 *
Mg^2+^	mg/L	1.9 ± 0.2	2.1 ± 0.3	2.0 ± 0.3	2.1 ± 0.2	2.2 ± 0.2	2.1 ± 0.1	2.2 ± 0.2	2.1 ± 0.1
Na^+^	mEq/dL	139.8 ± 3.8	141.6 ± 0.9	141.9 ± 1.4	142.4 ± 1.0 *	141.4 ± 1.1	140.7 ± 1.2	140.4 ± 1.2	141.0 ± 1.4
PO_4_^3−^	mg/dL	6.7 ± 1.0	6.7 ± 0.6	6.9 ± 1.0	7.4 ± 0.8	6.4 ± 0.9	5.8 ± 1.0	6.2 ± 0.8	5.7 ± 0.3

RQBACM: red quinoa and barley-based solid-state fermented AC mycelium ^1,2^ Values are presented as the mean ± SD, with *n* = 10 or *n* = 9 animals per group. ^3^ AST, aspartate aminotransferase; ALT, alanine aminotransferase; ALP, alkaline phosphatase; CK, creatine kinase; LDH, lactate dehydrogenase; GGT, gamma-glutamyltransferase; BUN, blood urea nitrogen; TB, total bilirubin; UA, uric acid; HDL-C, high-density lipoprotein cholesterol; TG, triglycerides; TP, total protein. * Statistically significant variance compared to the control group (*p* < 0.05).

**Table 6:** Absolute organ weight changes in rats treated with RQBACM in the 90-day feeding toxicity test.

Organ (g)	Male				Female			
		RQBACM (mg/kg)				RQBACM (mg/kg)		
	Control	1000	2000 ^2^	3000	Control	1000	2000	3000
Adrenal glands	0.057 ± 0.013 ^1^	0.057 ± 0.010	0.053 ± 0.011	0.061 ± 0.010	0.059 ± 0.008	0.069 ± 0.010	0.067 ± 0.008	0.066 ± 0.014
Brain	2.2 ± 0.1	2.2 ± 0.1	2.2 ± 0.1	2.2 ± 0.1	2.0 ± 0.1	2.0 ± 0.1	2.0 ± 0.1	2.0 ± 0.1
Heart	1.6 ± 0.1	1.5 ± 0.1	1.5 ± 0.1	1.5 ± 0.1	0.9 ± 0.1	0.9 ± 0.1	0.9 ± 0.1	0.9 ± 0.1
Kidney	3.6 ± 0.3	3.5 ± 0.3	3.5 ± 0.4	3.6 ± 0.3	1.8 ± 0.2	1.9 ± 0.3	1.8 ± 0.2	1.9 ± 0.2
Liver	13.9 ± 1.3	13.9 ± 2.0	14.2 ± 1.1	14.5 ± 1.8	6.5 ± 0.5	6.9 ± 1.1	6.8 ± 0.7	7.2 ± 1.1
Spleen	0.7 ± 0.1	0.7 ± 0.1	0.7 ± 0.1	0.7 ± 0.1	0.4 ± 0.0	0.4 ± 0.1	0.4 ± 0.1	0.4 ± 0.1
Thymus	0.4 ± 0.1	0.3 ± 0.1	0.3 ± 0.1	0.3 ± 0.1	0.2 ± 0.0	0.2 ± 0.0	0.2 ± 0.1	0.3 ± 0.1
Testis	3.2 ± 0.2	3.3 ± 0.2	2.9 ± 0.9	3.4 ± 0.2	-	-	-	-
Ovary	-	-	-	-	0.07 ± 0.01	0.07 ± 0.02	0.07 ± 0.02	0.07 ± 0.01

RQBACM: red quinoa and barley-based solid-state fermented AC mycelium. ^1^ Data are expressed as the mean ± *SD* (*n* = 10). ^2^ Data are expressed as the mean ± *SD* (*n* = 9). Significant difference between the control and treated groups at *p* < 0.05.

**Table 7:** Relative organ weight changes in rats treated with RQBACM in the 90-day feeding toxicity test.

Organ	Male				Female			
		RQBACM (mg/kg)				RQBACM (mg/kg)		
	Control	1000	2000 ^2^	3000	Control	1000	2000	3000
Adrenal glands (‰)	0.10 ± 0.02 ^1^	0.11 ± 0.02	0.10 ± 0.02	0.11 ± 0.02	0.22 ± 0.02	0.24 ± 0.03	0.24 ± 0.03	0.23 ± 0.04
Brain (%)	0.38 ± 0.03	0.41 ± 0.05	0.41 ± 0.03	0.40 ± 0.02	0.75 ± 0.05	0.70 ± 0.07	0.72 ± 0.06	0.70 ± 0.05
Heart (%)	0.27 ± 0.01	0.28 ± 0.02	0.28 ± 0.02	0.28 ± 0.02	0.32 ± 0.03	0.32 ± 0.02	0.32 ± 0.02	0.34 ± 0.03
Kidney (%)	0.62 ± 0.06	0.64 ± 0.06	0.65 ± 0.04	0.67 ± 0.04	0.64 ± 0.07	0.67 ± 0.03	0.63 ± 0.04	0.68 ± 0.06
Liver (%)	2.39 ± 0.13	2.56 ± 0.21	2.58 ± 0.19	2.68 ± 0.24 *	2.39 ± 0.16	2.38 ± 0.16	2.44 ± 0.14	2.52 ± 0.21
Spleen (%)	0.12 ± 0.01	0.13 ± 0.02	0.12 ± 0.01	0.13 ± 0.02	0.15 ± 0.02	0.15 ± 0.02	0.15 ± 0.02	0.15 ± 0.02
Thymus (%)	0.06 ± 0.01	0.06 ± 0.02	0.06 ± 0.01	0.06 ± 0.02	0.08 ± 0.01	0.07 ± 0.01	0.08 ± 0.02	0.09 ± 0.03
Testis (%)	0.56 ± 0.06	0.62 ± 0.07	0.54 ± 0.18	0.63 ± 0.04	-	-	-	-
Ovary (%)	-	-	-	-	0.02 ± 0.00	0.03 ± 0.01	0.03 ± 0.01	0.02 ± 0.00

RQBACM: red quinoa and barley-based solid-state fermented AC mycelium. ^1^ Data are expressed as the mean ± *SD* (*n* = 10). ^2^ Data are expressed as the mean ± *SD* (*n* = 9). * Significant difference between the control and treated groups at *p* < 0.05.

## Data Availability

The original contributions presented in this study are included in the article/Supplementary Materials. Further inquiries can be directed to the corresponding authors.

## Supplementary Materials

The following supporting information can be downloaded at: https://www.mdpi.com/article/10.3390/foods15152723/s1, Figure S1: Cytotoxicity of RQBACM co-incubated with CHO-K1 cells for 24 h; Figure S2: Feed efficiency of rats administered RQBACM in the 90-day feeding toxicity study; Table S1: Bacterial toxicity of RQBACM in Salmonella typhimurium TA98, TA100, TA102, TA1535, TA1537 tester strains; Table S2: Historical control range for spontaneous aberrations in CHO-K1 in our laboratory; Table S3: Clinical signs and body weight changes of mice after gavage treated with RQBACM in micronucleus test; Table S4: Summary of gross and histopathological findings in rats with observed lesions from the control and RQBACM-treated groups in the 90-day feeding study; Table S5: Summary of pathological incidence of rats in other organs in the 90 days oral toxicity of RQBACM.
