# Supplementary material for: Safety Assessment of Antrodia cinnamomea Cultured in Red Quinoa and Barley: Genotoxicity in Mice and 90-Day Feeding Toxicity in Rats
Source: Foods. 2026 Aug 3;15(15):2723. doi: 10.3390/foods15152723 (PMC13464894; doi:10.3390/foods15152723)
Supplement: Supplementary file 1 [file foods-15-02723-s001.zip › foods-4345135-supplementary.pdf]

Supplementary Information

Figure S1.Cytotoxicity of RQBACM co-incubated with CHO-K1 cells for 24 hours

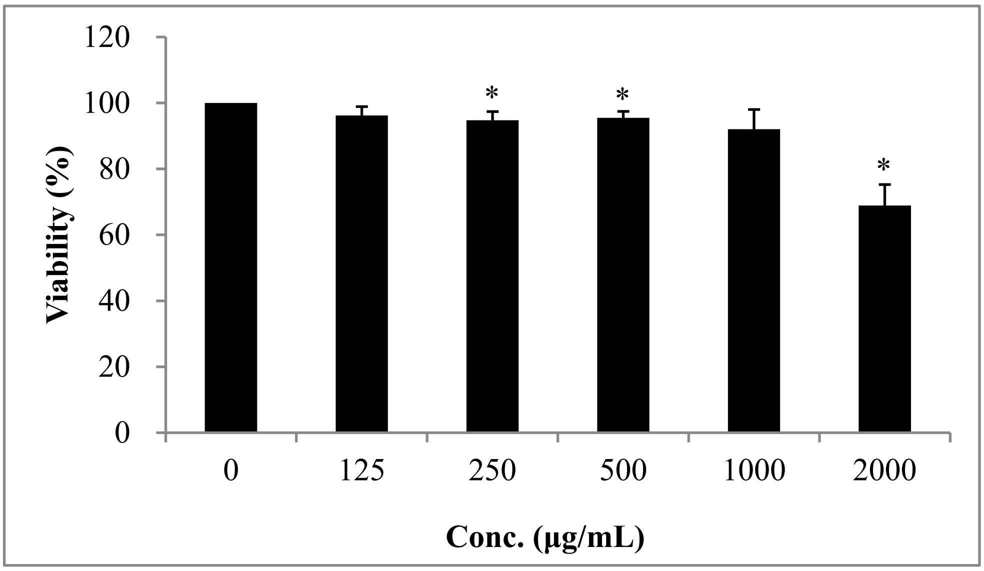

Figure S2.Feed efficiency of rats administered RQBACM in the 90-day feeding toxicity study

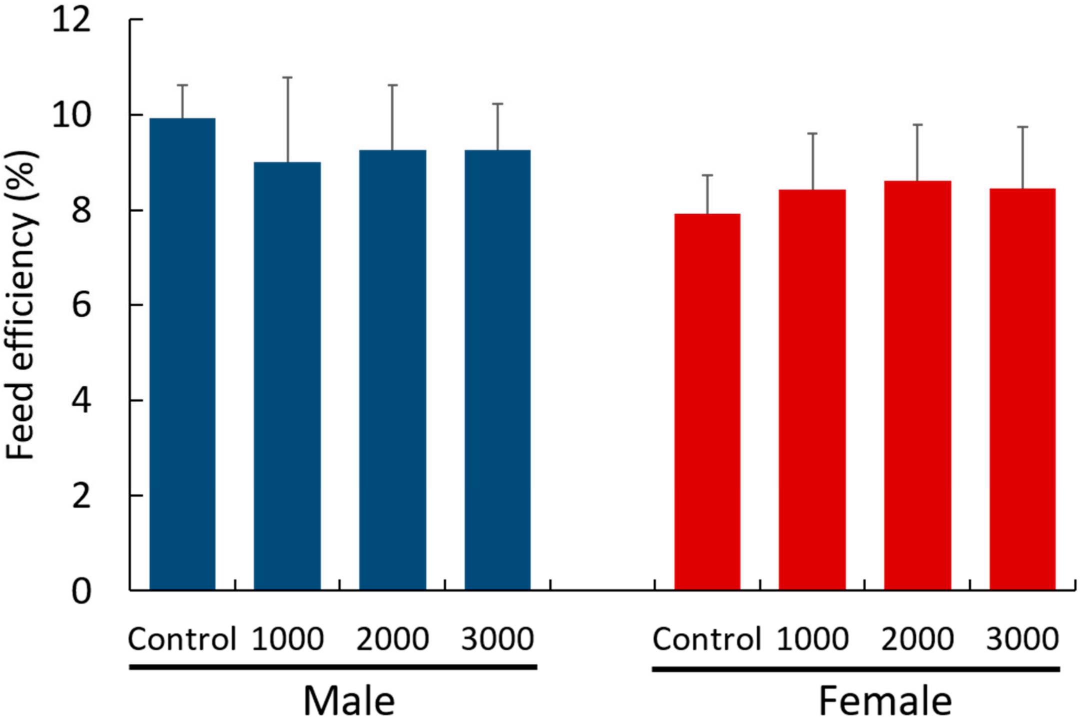

Table S1. Bacterial toxicity of RQBACM in *Salmonella typhimurium* TA98, TA100, TA102, TA1535, TA1537 tester strains

Tester strains	Dilution folds	Conc. (μg/plate)/Number of revertants (colony, CFU/plate)			
		0	1,250	2,500	5,000
TA98					
	10^6	124.7 ± 6.8	126.3 ± 4.1	127.7 ± 3.9	116.7 ± 7.6
	10^7	15.0 ± 2.4	13.3 ± 1.2	15.7 ± 3.8	15.7 ± 1.7
TA100					
	10^6	165.0 ± 2.9	159.7 ± 3.4	152.7 ± 8.3	156.0 ± 4.1
	10^7	15.0 ± 2.9	16.0 ± 2.9	16.7 ± 1.2	13.7 ± 2.5
TA1535					
	10^6	248.0 ± 9.2	242.7 ± 4.2	235.3 ± 3.7	228.7 ± 8.4
	10^7	26.7 ± 2.1	25.7 ± 0.5	24.3 ± 2.5	24.0 ± 3.3

Tester strains	Dilution folds	Conc. (μg/plate)/Number of revertants (colony, CFU/plate)			
		0	1,250	2,500	5,000
TA1537					
	10^6	116.0 ± 6.2	104.7 ± 1.7	88.7 ± 13.5	70.7 ± 7.0*
	10^7	10.0 ± 1.4	6.3 ± 4.5	9.0 ± 2.2	4.3 ± 1.9*
TA102					
	10^6	309.3 ± 12.5	300.0 ± 6.4	296.0 ± 6.4	261.3 ± 8.2*
	10^7	32.7 ± 0.9	33.3 ± 0.5	30.0 ± 2.2	26.7 ± 2.1*

Data are expressed as the mean ± SD (n = 3).

* Significant difference of colonies more than two folds of negative control and treated groups at $p < 0.05$.

Table S2. Historical control range for spontaneous aberrations in CHO-K1 in our laboratory

Study	Date	% Aberration (negative control)
Study 1	2017	3.0 ± 2.6
Study 2	2019	5.7 ± 3.2
Study 3	2019	4.0 ± 1.0
Study 4	2020	3.7 ± 1.5
Study 5	2021	2.7 ± 1.5

Table S3. Clinical signs and body weight changes of mice after gavage treated with RQBACM in micronucleus test

Group	Clinical observation	Body weight (g)		
		Day 0	Day 2	Day 3
Male				
Negative control	Normal	32.4 ± 1.7	34.5 ± 1.9	34.5 ± 1.6
Positive control[2]	Normal	34.1 ± 2.0	35.7 ± 2.2	35.3 ± 1.9
RQBACM				
1,000 mg/kg	Normal	33.7 ± 1.7	35.6 ± 1.8	35.3 ± 1.7
3,000 mg/kg	Normal	33.1 ± 1.6	35.1 ± 2.0	35.1 ± 2.4
5,000 mg/kg	Normal	34.1 ± 2.7	35.6 ± 2.3	35.7 ± 2.4

Control: Reverse osmosis water

[1] Data are expressed as the mean ± SD (n = 5).

[2] Positive control: Cyclophosphamide 60 mg/kg bw. ip

Table S4. Summary of gross and histopathological findings in rats with observed lesions from the control and RQBACM-treated groups in the 90-day feeding study

Sex/Group	No. of Animals	Organ	Gross findings	Microscopic findings
Male				
Control	10	All	No significant lesion	-
1,000 mg/kg	10	All	No significant lesion	-
2,000 mg/kg	7	All	No significant lesion	-
	1	Testes	Bilateral atrophy (ID: 304)	Atrophy, seminiferous tubule, diffuse, severe/high[1]
	1	Lungs	Multifocal erythema (ID: 306)	No significant change
3,000 mg/kg	10	All	No significant lesion	-
Female				
Control	10	All	No significant lesion	-
1,000 mg/kg	10	All	No significant lesion	-
2,000 mg/kg	10	All	No significant lesion	-
3,000 mg/kg	9	All	No significant lesion	-
	1	Kidney	Left kidney enlargement (ID: 411)	Pyelonephritis, diffuse, moderate/severe

-: No significant lesions.

[1]: Degree of lesions was graded from one to five depending on severity: 1 = minimal (< 1%); 2 = slight (1-25%); 3 = moderate (26-50%); 4 =moderate/severe (51-75%); 5 = severe/high (76-100%).

Table S5. Summary of pathological incidence of rats in other organs in the 90 days oral toxicity of RQBACM

Organ	Lesions	Group			
		Control		High dose	
		Male	Female	Male	Female
Adrenal gland		-	-	-	-
	Hypertrophy, cholesterol, zona fasciculata, focal, slight to moderate[1]	7/10[2]	4/10	9/10	4/10
	Lipidosis, cytoplasmic, zona fasciculata, focal, slight to moderate	2/10	-	4/10	-
Aorta		-	-	-	-
Brain					
Fore		-	-	-	-
Middle		-	-	-	-
Cerebellum		-	-	-	-
Stem		-	-	-	-
Bone, femur		-	-	-	-
Bone marrow		-	-	-	-
Esophagus		-	-	-	-
Eyes		-	-	-	-
Harderian gland					
	Infiltration, mononuclear cell, focal, minimal to slight[1]	3/10[2]	-	-	1/10
Heart					
	Infiltration, mononuclear cell, focal, slight	2/10	-	-	-
Intestine, small					
Duodenum		-	-	-	-
Jejunum		-	-	-	-
Ileum		-	-	-	-
Intestine, large					
Caecum		-	-	-	-
Colon		-	-	-	-
Rectum		-	-	-	-
Kidney					
	Cyst, tubule, focal, minimal	1/10	-	-	-
	Infiltration, mononuclear cell, multifocal, minimal to slight	2/10		1/10	-
	Pyelonephritis, unilateral, diffuse, moderate/severe	-	-	-	1/10
	Regeneration, tubule, multifocal, minimal to slight	2/10	-	1/10	-
Liver		-	-	-	-
Lung		-	-	-	-
Lymph node					
Cervical		-	-	-	-
Mesenteric		-	-	-	-
Mammary gland		N	-	N	-

Organ	Lesions	Group			
		Control		High dose	
		Male	Female	Male	Female
Optic nerve		-	-	-	-
Ovary	Hypertrophy, cholesterol, interstitial cell, slight	-	2/10	-	2/10
Pancreas					
	Atrophy, glandular, focal, minimal to slight	2/10	-	1/10	-
	Fibrosis, islet, multifocal, slight	4/10	-	1/10	-
	Pigmentation, islet, multifocal, minimal	2/10	-	1/10	-
	Infiltration, mononuclear cell, focal, slight	1/10		1/10	
Pituitary		-	-	-	-
Parathyroid gland		-	-	-	-
Prostate gland					
	Infiltration, mononuclear cell, multifocal, slight to moderate	2/10	N	1/10	N
Salivary gland					
Mandibular lobe		-	-	-	-
Sublingual lobe		-	-	-	-
Sciatic nerve		-	-	-	-
Seminal vesicle		-	N	-	N
Skeletal muscle		-	-	-	-
Skin		-	-	-	-
Spinal cord					
Cervical		-	-	-	-
Lumbar		-	-	-	-
Thoracic		-	-	-	-
Spleen		-	-	-	-
Stomach		-	-	-	-
Testes		-	N	-	N
Epididymis		-	N	-	N
Thymus		-	-	-	-
Thyroid gland		-	-	-	-
Tongue		-	-	-	-
Trachea		-	-	-	-
Urinary bladder		-	-	-	-
	Infiltration, mononuclear cell, multifocal, slight	-	-	-	1/10
	Hyperplasia, epithelial, diffuse, slight	-	-	-	1/10
Uterus		N	-	N	-
Vagina		N	-	N	-

Control: D.W. (10 mL/kg); High dose: 3,000 mg/kg RQBACM.
- No effect. N: no tissue available.
[1] Degree of lesions was graded from one to five depending on severity: 1 = minimal (< 1%); 2 = slight (1-25%); 3 = moderate (26-50%); 4 = moderate/severe (51-75%); 5 = severe/high (76-100%).
[2] Incidence: Affected rats/ Total examined rats (n = 10).
